# Self-frequency-conversion nanowire lasers

**DOI:** 10.1038/s41377-022-00807-7

**Published:** 2022-04-29

**Authors:** Ruixuan Yi, Xutao Zhang, Chen Li, Bijun Zhao, Jing Wang, Zhiwen Li, Xuetao Gan, Li Li, Ziyuan Li, Fanlu Zhang, Liang Fang, Naiyin Wang, Pingping Chen, Wei Lu, Lan Fu, Jianlin Zhao, Hark Hoe Tan, Chennupati Jagadish

**Affiliations:** 1 Key Laboratory of Light Field Manipulation and Information Acquisition, Ministry of Industry and Information Technology, and Shaanxi Key Laboratory of Optical Information Technology, School of Physical Science and Technology, Northwestern Polytechnical University, 710129 Xi’an, China; 2 Frontiers Science Center for Flexible Electronics, Xi’an Institute of Flexible Electronics (IFE) and Xi’an Institute of Biomedical Materials & Engineering, Northwestern Polytechnical University, 127 West Youyi Road, 710072 Xi’an, China; 3 Department of Electronic Materials Engineering, Research School of Physics, The Australian National University, Canberra, ACT 2601 Australia; 4 State Key Laboratory for Infrared Physics, Shanghai Institute of Technical Physics, Chinese Academy of Sciences, 500 Yutian Road, 200083 Shanghai, China; 5 University of Chinese Academy of Sciences, 19 Yuquan Road, 100049 Beijing, China; 6 School of Physical Science and Technology, ShanghaiTech University, 393 Middle Huaxia Road, Pudong District, 201210 Shanghai, China; 7 ARC Centre of Excellence for Transformative Meta-Optical Systems, Research School of Physics, The Australian National University, Canberra, ACT 2601 Australia

**Keywords:** Semiconductor lasers, Nanowires, Nonlinear optics

## Abstract

Semiconductor nanowires (NWs) could simultaneously provide gain medium and optical cavity for performing nanoscale lasers with easy integration, ultracompact footprint, and low energy consumption. Here, we report III–V semiconductor NW lasers can also be used for self-frequency conversion to extend their output wavelengths, as a result of their non-centrosymmetric crystal structure and strongly localized optical field in the NWs. From a GaAs/In_0.16_Ga_0.84_As core/shell NW lasing at 1016 nm, an extra visible laser output at 508 nm is obtained via the process of second-harmonic generation, as confirmed by the far-field polarization dependence measurements and numerical modeling. From another NW laser with a larger diameter which supports multiple fundamental lasing wavelengths, multiple self-frequency-conversion lasing modes are observed due to second-harmonic generation and sum-frequency generation. The demonstrated self-frequency conversion of NW lasers opens an avenue for extending the working wavelengths of nanoscale lasers, even to the deep ultraviolet and THz range.

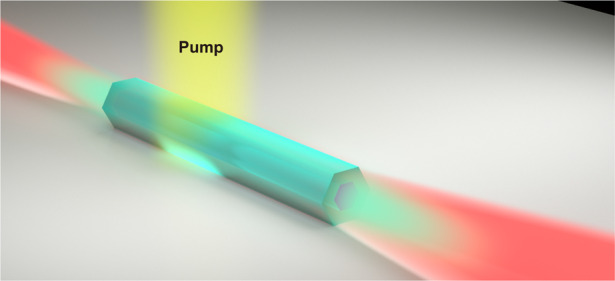

## Introduction

Nanowire (NW) lasers are attractive for constructing miniaturized photonic integration systems owing to their merits of ultracompact footprint, easy integration, low threshold, and low energy consumption. Remarkably, semiconductor NW simultaneously functions as the gain medium and optical cavity of a nanoscale laser, enabling its easy fabrication, operation, and potentially large-scale production^1–7^. NW lasers have been demonstrated in a variety of materials, such as III–V and II–VI compound semiconductors, and perovskites^3,8–16^. With the successful introduction of quantum confined structures such as quantum dots and quantum wells into NWs, the gain characteristics could be flexibly tuned and NW lasers exhibit superior device performance, like low threshold and high-temperature stability^10,11,17–19^. In recent years, the development of selective area epitaxy enables the demonstration of NW-array-based lasers with other photonic modes^6,20,21^. These achievements greatly promote the potential of using NW lasers in applications including optical interconnects, sensing, displaying, and microscopy. Unfortunately, limited by the specific gain spectral range of the semiconductor NWs, there is still some wavelength range that cannot be easily obtained from NW lasers, thereby narrowing their application areas. While the emission peaks of compound semiconductor NWs could be theoretically shifted in a moderately broad range by controlling the alloy composition, there are many material-related challenges to the growth of NWs over a broad compositional range. For example, due to the high density of dislocations of the quantum well with high indium content, green InGaN NW lasers are still not realized^10,22^.

In the development history of commercial lasers employed in laboratories and industry, such as high-power solid-state lasers, gas lasers, and fiber lasers, the laser operation wavelength range is also limited by the gain medium. One of the widely used solutions to convert the lasing wavelength is to utilize an external nonlinear optical crystal to achieve nonlinear optical parametric conversions^23^. For example, the high-power green laser at the wavelength of 532 nm is typically realized by frequency-doubling the Nd:YAG laser at 1064 nm via the second-harmonic generation (SHG). In addition, lasers with continuously tuned wavelengths are normally achieved by optical parametric amplifiers or optical parametric oscillators using crystals with second-order nonlinearity. These successes inspire the utilization of nonlinear frequency conversion to extend the output wavelength range of NW lasers. However, due to the low output power of NW lasers, it is not practical to employ NW lasers as the source to pump another nonlinear crystal to achieve frequency conversion.

In this work, we show that the intrinsic second-order nonlinearity of semiconductor NWs can be used to convert the wavelength of the NW laser by itself. For NW lasers based on III–V and II–V compound semiconductors, such as GaAs, InP, CdS, CdTe, their crystals have no inversion symmetry and therefore support intrinsic second-order nonlinearity^24–27^. Compared with the widely employed nonlinear crystals in laser technology, such as BBO, KDP, KTP, these semiconductor NWs typically have one or two orders of magnitude higher second-order nonlinearities, which could significantly facilitate the self-frequency-conversion in their NW lasers^23^. On the other hand, in a NW laser, the fundamental laser mode is strongly confined within its cross-section with a diameter of a few hundred nanometers, which provides a significantly high density of the optical field. As a consequence, the second-order nonlinear optical processes could be self-realized effectively. In this report, we grew high-quality GaAs/In_0.16_Ga_0.84_As core/shell NWs and demonstrate lasing at the wavelength of 1016 nm. In addition to this fundamental wavelength, a visible laser output is also obtained at 508 nm via doubling the frequency of the fundamental lasing mode, i.e., the SHG process. These two modes are analyzed by their far-field polarization dependence and the second-order nonlinear susceptibility tensor of the zincblende (ZB) crystal structure of the NWs. These NW lasers can also operate in multimode via SHG and sum-frequency generation (SFG). The proposed self-frequency-conversion NW lasers provide a solution for expanding the working wavelength of these lasers, potentially finding applications in optical parametric generators, amplifiers, and oscillators operating over a large wavelength range from deep ultraviolet to THz.

## Results

**Fig. 1:**
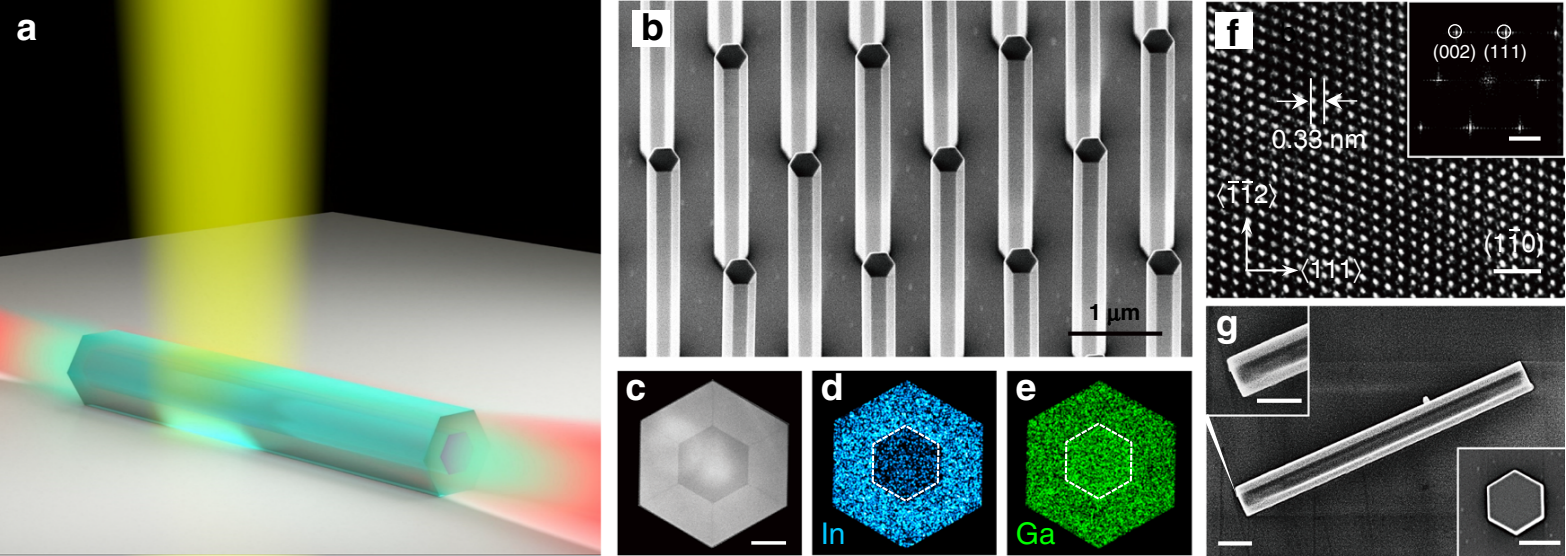
Schematic illustration of the self-frequency conversion NW laser and its structural properties. **a** The schematic of NW laser with fundamental lasing (red light) and self-frequency-conversion lasing (cyan light) signals pumped by an 800 nm femto-second pulsed laser (yellow light). **b** Tilted 30° SEM image of the as-grown NW array. **c–e** HAADF-STEM (**c**), In (**d**) and Ga (**e**) mapping images of the NW cross-section. Scale bar is 100 nm. **f** HRTEM and FFT (inset) images taken along $\langle1\bar 10\rangle$ zone axis of the NW showing a zincblende crystal structure. Scale bar is 1 nm. Scale bar of inset is 2 nm^−1^. **g** SEM image of a NW transferred onto a SiO_2_/Si substrate. Upper-left inset and bottom-right inset show the zoomed images of the end facet of the transferred NW and end facet of a standing NW, respectively, indicating the high facet quality of the NW as an optical cavity. All scale bars are 500 nm

The self-frequency-conversion NW laser is schematically shown in Fig. 1a. The NWs were grown by selective area epitaxy (SAE) method using metal-organic chemical vapor deposition (MOCVD) (more details in Supplementary information Section 1). Figure 1b shows the scanning electron microscope (SEM) image of the as-grown NW array standing on the GaAs substrate. The NWs have a uniform hexagonal prism cross-section and consist of a core/shell GaAs/In_x_Ga_1−x_As structure. To reveal the details of this heterostructure, we performed transmission electron microscopy (TEM) studies on the cross-section of the NW, which is prepared by the focused ion beam technique. Figure 1c shows the high-angle annular dark-field scanning transmission electron microscopy (HAADF-STEM) image of the cross-sectional sample. The darker and brighter regions correspond to a 210 nm-thick GaAs core and 110 nm-thick In_x_Ga_1−x_As shell, respectively. Figure 1d and e show the In and Ga energy-dispersive X-ray spectroscopy (EDS) mapping of the cross-sectional sample, confirming the core/shell structure with a Ga/In the ratio of 0.84/0.16 in the shell region. As the shell region has a narrower bandgap than the core, carriers will be confined in the shell region and provide higher optical gain for high-order transverse mode with donut-like intensity distribution compared to the fundamental transverse mode^28^. The distribution of the SHG radiation sources of the donut-like mode is closer to the surface of NW than that of the fundamental mode, which means less reabsorption inside the NW and higher radiation efficiency outside the NW of the SHG lasing mode. Therefore, the core/shell structure NWs were selected (see details in Supplementary information Section 9). Figure 1f shows the high-resolution transmission electron microscopy (HRTEM) and the corresponding fast Fourier transformation (FFT) images taken along $\langle1\bar 10\rangle$ zone axis of the NW. The NWs were determined to have a ZB crystal phase and with the side facets being as $\left\{ {110} \right\}$. The crystal orientation will be discussed later. In the implementation of the NW laser, the NW is separated from the growth substrate and placed horizontally on a SiO_2_(285 nm)/Si substrate. The optical cavity of the laser is formed by the two end facets of the NW, which function as two parallel mirrors and form a Fabry–Pérot cavity. A SEM image of a NW lying horizontally on the SiO_2_/Si substrate is shown in Fig. 1g, where the insets display the end facets. The flat end facets of the NW are perpendicular to the longitudinal direction, which enables high reflectance for the guided mode in the NW as well as resonance modes of the optical cavity with high-quality factors ^19^.

The lasing behavior of the NW was characterized in a home-built confocal microscope system, where the NW is placed in a cryostat (see Materials and methods). Figure S1 in the Supplementary information Section 2 shows the schematic sketch of the experimental setup. In particular, the setup could separate the fundamental lasing light and self-frequency-conversion lasing light in space through a dichroic mirror and analyze them simultaneously and independently. To facilitate the analysis of the second-order nonlinear process in the NW, we defined the coordinate system of the lab frame and the NW orientation, as shown in Supplementary information Fig. S1. According to the crystal structure revealed by the TEM measurements, the *x*, *y*, *z* axes correspond to the ZB crystal direction $\langle111\rangle$, $\langle\bar 1\bar 12\rangle$ and $\langle1\bar 10\rangle$, respectively^29^.

**Fig. 2:**
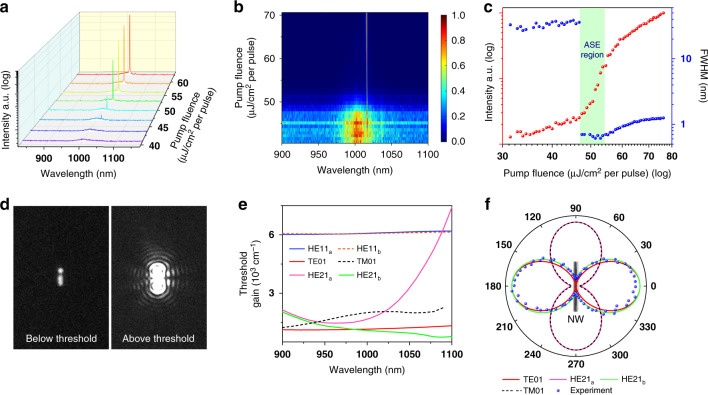
Lasing characteristics and mode identification of the NW laser. **a, b** Emission spectra of the NW with increasing pump fluences (**a**) and the normalized spectra map (**b**). **c** Integrated lasing peak intensity (red dots) and the corresponding FWHM (blue dots) of the emission spectra versus pump fluence on a log–log plot. The ASE region is highlighted by the light green shaded area. **d** Optical images of the NW emission below (left) and above (right) the lasing threshold. **e** Calculated threshold gain spectra for supported guided modes of the core/shell NW with a diameter and length of 410 nm and 4.4 μm, respectively. **f** Polarization dependence plots of possible lasing modes calculated from 3D FDTD simulations and the experimentally measured data

The lasing behavior of a GaAs/In_0.16_Ga_0.84_As core/shell NW with a diameter of 410 nm and length of 4.4 μm was first characterized. The emission spectra from the NW at various pump fluences are presented in Fig. 2a, b. At very low pump fluence (<31.3 μJ/cm^2^/pulse), the photoluminescence (PL) spectrum has a broad single peak centered at 1010 nm with a full width at half maximum (FWHM) of 50 nm, corresponding to the spontaneous emission from the In_0.16_Ga_0.84_As shell. At a pump fluence of ∼44 μJ/cm^2^/pulse, a small peak at 1016 nm appears in the spectrum. This peak becomes more pronounced with increasing pump fluence and can be ascribed to amplified spontaneous emission (ASE) in the NW. At a pump fluence of ∼54.5 μJ/cm^2^/pulse, the intensity of the cavity peak increases rapidly, with a measured linewidth as narrow as FWHM = 0.8 nm. Figure 2c presents the extracted integrated intensity of this peak and its FWHM versus pump fluence from Fig. 2a. The “S”-shaped the nonlinear response of the lasing mode on the log–log scale and sharp transition in the FWHM above a pump threshold is clearly observed^30^. Figure 2d shows the optical images of the NW emission below and above the lasing threshold. Compared with the image acquired below the threshold, the image obtained above the threshold presents bright emission from the NW ends and a distinct interference pattern^31^. All these features conclusively indicate that lasing occurs in the NW.

For this NW with a relatively large diameter of 410 nm, there are several possible resonance modes supported by the NW optical cavity. To facilitate the following analysis of the frequency-doubled lasing mode from the NW laser, it is necessary to determine the details of the modes supported in this cavity, including the electric field distribution, and polarization of the far-field radiation. Figure 2e shows the mode identification of the NW laser with a combination of lasing threshold calculations for each mode.

In the numerical calculations, we use a three-dimensional model of core/shell NW lying on a SiO_2_(285 nm)/Si substrate. Considering the high refractive index of the GaAs NW, there are 6 transverse modes that could be supported, which are the HE11_a/b_, TE01, TM01, and HE21_a/b_. Their threshold gain spectra *g*_th_ could be calculated by

1 $$g_{{{{\mathrm{th}}}}} = \frac{1}{{\Gamma }}\left( {\frac{1}{L}\ln \frac{1}{R} + \alpha _i} \right)$$

in which *L* is the length of NW, Γ is the mode confinement factor, *R* is the mode facets reflectivity and *α*_i_ is the mode propagation losses^28,32^. Mode confinement factor was calculated by a 2D finite-difference eigenmode (FDE) solver. As the diameter of NW is typically smaller than the lasing wavelength, the calculation of facet reflectivity involves diffraction and couldn’t be solved by the Fresnel formula^33^. A 3D finite-difference time-domain (FDTD) method was used to calculate the facet reflectivity for each mode. And the propagation losses were also calculated by this method (see Supplementary information Section 3). The possible lasing modes could arise from the modes with lower threshold gains, such as HE21_a_, HE21_b_, TE01, and TM01. The threshold gains of the modes HE11_a_, HE11_b_ are much higher than others, and therefore considered unlikely to lase in our NW.

To further identify the experimentally observed lasing mode of the NW, its polarization characteristic of the far-field radiation was measured by rotating the linear polarizer (LP) shown in Supplementary information Fig. S1. The polarization-dependent intensities of the dominant lasing peak with a pump fluence of 54.5 μJ/cm^2^/pulse are shown in Fig. 2f. The orientation of the NW is also schematically shown in the polar plot. The laser emission is polarized perpendicular to the long axis of the NW and has a polarization ratio, *ρ* = (*I*_∥_ − *I*_⊥_)/(*I*_∥_ + *I*_⊥_), of 0.768, which clearly indicates the amplification of a particular transverse mode in the NW cavity. The far-field emission profiles of the theoretically obtained possible lasing modes HE21_a_, HE21_b_, TE01, and TM01 were numerically calculated^34^, which could represent their far-field polarization dependences (see the Supplementary information Section 7.1). As shown in Fig. 2f, only the polarization dependence of the HE21_b_ mode (both polarization orientation and ratio) matches with the experimental data, which indicates the lasing mode is the HE21_b_ mode. Note, though the TE01 mode has the same polarization orientation as that of the experiment results, the extinction ratios have a large deviation between the simulation and experiment.

**Fig. 3:**
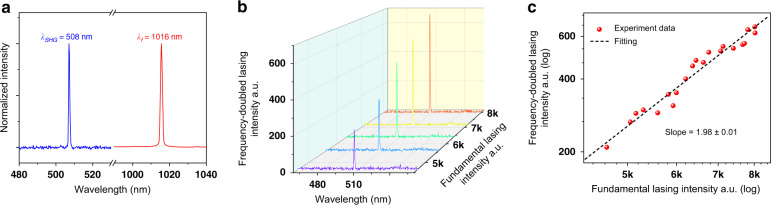
Characteristic of the frequency-doubled lasing mode of the NW laser via the SHG process. **a** Normalized spectra of the fundamental lasing mode (1016 nm) and the frequency-doubled lasing mode (508 nm). **b** Emission spectra of the frequency-doubled lasing mode with the increasing fundamental lasing intensity. **c** Dependence of the fundamental lasing mode and the frequency-doubled lasing mode intensities, showing a fitting slope 1.98 ± 0.01 on a log–log scale

After the characterization of the fundamental lasing mode of the core/shell NW, the emission behavior at the wavelength range shorter than the pump wavelength (of 800 nm) was also monitored. At the low pump fluences (<50 μJ/cm^2^/pulse), only a very weak peak at the wavelength of 400 nm is observed, which is due to the SHG signal of the pump laser (Supplementary information Fig. S8a). By further increasing the pump fluence gradually, a signal peak at the wavelength of 508 nm emerges, as shown in Fig. 3a. Considering the fundamental lasing mode of the NW at 1016 nm, this peak should be the SHG pumped by the lasing mode directly. Because the second-order nonlinearity of the GaAs is driven by electric-dipole polarization, the SHG process is instantaneous, which promises the SHG signal maintains the coherence of the pump laser^35^. Hence, the visible laser mode at 508 nm could be considered a frequency-doubled lasing mode of the NW. To further confirm this, the function between the laser intensity at 508 nm and the laser intensity at 1016 nm was examined and the emission spectra are shown in Fig. 3b. The extracted peak intensities from Fig. 3b are plotted in Fig. 3c on a log–log scale. The measurement results can be fitted by a line with a slope of 1.98 ± 0.01, proving that the visible lasing mode results from the second-order nonlinear process of the fundamental near-infrared lasing mode.

**Fig. 4:**
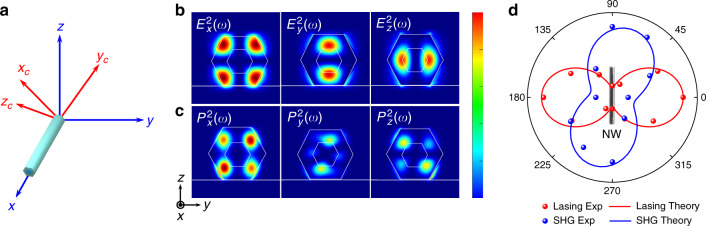
Theoretical modeling and experimental results of the frequency-doubled mode. **a** Schematic of the lab frame (*xyz*) and the crystal frame (*x*_*c*_*y*_*c*_*z*_*c*_). **b** Electric field distribution of the HE21_b_ mode of the NW in the *yz* plane, and its *x*, *y*, and *z* components. **c** Calculated second-order nonlinear polarization according to the electric field of the HE21_b_ mode and second-order nonlinear susceptibility tensor of the GaAs. **d** Polarization dependence of the frequency-doubled lasing mode (blue dots) and its theoretical prediction (blue line), where the corresponding polarization dependence of the fundamental lasing mode is also shown (red dots for experimental results and red line for theoretical calculation)

Due to the strong absorption coefficient of GaAs around the wavelength of 500 nm (~10.7 μm^−1^)^36^, the axial propagation of SHG would not form a guiding mode. The measured SHG signal originates from the radiation of oscillating second-order nonlinear polarizations which directly pass through the NW surface in the normal direction (see details in Supplementary information Section 9). To confirm the mechanism of SHG, we built a theoretical model to describe the second-order nonlinear process in the NW and verified it through a far-field polarization dependence experiment. Determined by the second-order nonlinear susceptibility tensor of the GaAs/In_0.16_Ga_0.84_As, it is necessary to construct the model by considering the relation between the crystal structure frame and the lab frame, as shown in Fig. 4a. The ZB structure of GaAs belongs to $\bar 43m$ classes of points group, which has non-zero second-order nonlinear susceptibility components, *d*_14_ = *d*_25_ = *d*_36_ = 370 pm/V^23^. Considering the similar crystal structure and second-order nonlinear susceptibility tensor of pure GaAs and InAs, the alloy-induced changes of second-order nonlinear susceptibility in InGaAs are ignored. The SHG response of the NW can be calculated by its nonlinear polarization,

2 $${{{\mathbf{P}}}}_{{{\boldsymbol{c}}}} = 2\varepsilon _0\left[ {\begin{array}{*{20}{c}} 0 & 0 & 0 & {d_{14}} & 0 & 0 \\ 0 & 0 & 0 & 0 & {d_{25}} & 0 \\ 0 & 0 & 0 & 0 & 0 & {d_{36}} \end{array}} \right]\left[ {\begin{array}{*{20}{c}} {E_{cx}^2} \\ {E_{cy}^2} \\ {E_{cz}^2} \\ {2E_{cy}E_{cz}} \\ {2E_{cx}E_{cz}} \\ {2E_xE_y} \end{array}} \right]$$

in which *E*_*cx*_, *E*_*cy*_, and *E*_*cz*_ are the electric field components defined under the crystal frame. The excited electric field (*E*_*x*_, *E*_*y*_, *E*_*z*_) in the lab frame can then be transformed by a Euler matrix **R** into the crystal frame as *E*_*cx*_, *E*_*cy*_, and *E*_*cz*_ to calculate the nonlinear polarization **P**_*c*_ in the crystal frame according to:

3 $$\left[ {\begin{array}{*{20}{c}} {E_{cx}} \\ {E_{cy}} \\ {E_{cz}} \end{array}} \right] = {{{\mathbf{R}}}}\left[ {\begin{array}{*{20}{c}} {E_x} \\ {E_y} \\ {E_z} \end{array}} \right]$$

where the Euler matrix **R** is described in Supplementary information Section 4 and represents transformation between the crystal frame and the lab frame. Because the SHG signal from the NW laser is collected under the lab frame, the generated second-order nonlinear polarization **P**_*c*_ needs to be transformed to the lab frame as **P** through the inverse of Euler matrix **R**^−1^.

4 $$\left[ {\begin{array}{*{20}{c}} {P_x} \\ {P_y} \\ {P_z} \end{array}} \right] = {{{\mathbf{R}}}}^{ - 1}\left[ {\begin{array}{*{20}{c}} {P_{cx}} \\ {P_{cy}} \\ {P_{cz}} \end{array}} \right]$$

The second-order nonlinear polarization **P** could be regarded as electric dipoles that oscillate at SHG frequency and emit SHG signals into free space. According to the previous discussion, the fundamental lasing mode of the NW laser at the wavelength of 1016 nm was determined as HE21_b_. Through a FDTD simulation, the electric field distributions of this mode HE21_b_ in the *yz* plane are calculated, as shown in Fig. 4b. By substituting these components of the electric field into Eqs. (3), (2), and (4), the corresponding three components of second-order nonlinear polarization could be obtained numerically, as shown in Fig. 4c. These components of nonlinear polarizations account for the emission of the frequency-doubled lasing mode at the wavelength of 508 nm.

In the experiment, because the employed objective lens has a low numerical aperture, it has a very low collection efficiency of the SHG emission arising from the out-of-plane component (*P*_*z*_) of the second-order nonlinear polarization (see details in Supplementary information Section 5). The measured SHG signal is therefore predominantly contributed by the in-plane component (*P*_*x*_ and *P*_*y*_) of **P**, which gives rise to

5 $$I_{{{{\mathrm{SHG}}}}} \propto (P_x^2 + P_y^2)$$

the theoretical polarization dependence of the frequency-doubled mode could be calculated through integration as follows:

6 $$I(\theta) \propto \mathop{{\iint}\int}\limits_{{{{\mathrm{NW}}}}} {\frac{1}{V}\left( {P_x\left( {2\omega } \right)\cos \left(\theta \right) + P_y(2\omega)\sin \left(\theta \right)}\right)^2dv}$$

in which *θ* is the angle between NW long axis and the polarization, *V* is the volume of NW, and *dv* is the differential volume.

By rotating the linear polarizer in the setup shown in Supplementary information Fig. S1, the polarization dependence of the fundamental lasing mode and the frequency-doubled lasing mode are acquired, as plotted in Fig. 4d. The red dots are the measured results of the fundamental lasing mode with the theoretical fitting curve as the red solid line, which is the same as those shown in Fig. 2d. Correspondingly, the intensity variations of the frequency-doubled lasing mode with different polarizations are recorded, as shown by the blue dots in Fig. 4d. The measured results could be fitted well by the theoretical prediction governed by Eq. (6), as shown by the blue solid line of Fig. 4d. The fitting result has an angle of *θ* = 70°, which is determined by the electric field distribution of mode HE21_b_ and the NW crystal structure. It indicates that the obtained fundamental lasing mode and its self-frequency-doubled lasing mode have different polarizations, which facilitate the separation of the two laser wavelengths in future applications.

**Fig. 5:**
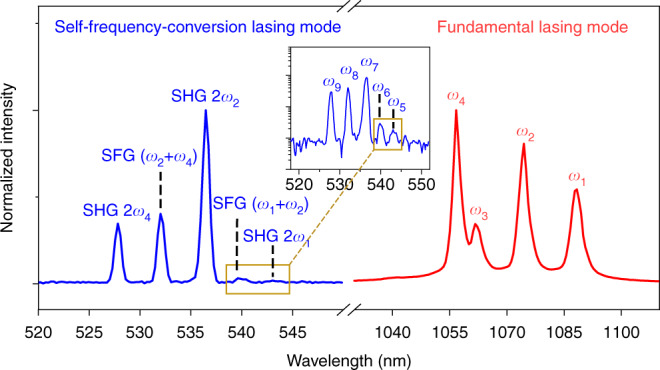
Multiple self-frequency-conversion wavelength output of a multimode NW laser due to second-order nonlinear effect. Normalized fundamental lasing modes (red line) and self-frequency-conversion modes (blue line), including SHG and SFG. Inset shows self-frequency-conversion signals plotted on logarithmic scale

Besides SHG, the second-order nonlinear effect could also support a variety of other three-wave-mixing processes, such as sum-frequency generation (SFG), difference-frequency generation (DFG), and spontaneous parametric downconversion (SPDC). The proposed self-frequency conversion of the NW laser for extending the output wavelength could also employ these three-wave-mixing processes. As an example, we use another GaAs/In_0.16_Ga_0.84_As core/shell NW with a diameter of 520 nm and 3.4 μm in length. As shown in Fig. 5, because of the high gain provided by the NW and the larger diameter, there are multiple fundamental lasing modes located at the wavelengths of *λ*_1_ = 1088 nm (*ω*_1_), *λ*_2_ = 1074 nm (*ω*_2_), *λ*_3_ = 1062 nm (*ω*_3_), *λ*_4_ = 1057 nm (*ω*_4_). In visible wavelength range in Fig. 5, multiple lasing peaks are observed at the wavelengths of *λ*_5_ = 544 nm (*ω*_5_), *λ*_6_ = 540.5 nm (*ω*_6_), *λ*_7_ = 537 nm (*ω*_7_), *λ*_8_ = 532.7 nm (*ω*_8_), and *λ*_9_ = 528.5 nm (*ω*_9_). According to the calculation of the frequency conversion, the lasing modes at these wavelengths could be directly correlated to the SHG and SFG signals of the fundamental lasing modes. *λ*_5_ (544 nm), *λ*_7_ (537 nm), and *λ*_9_ (528.5 nm) are generated from the SHG (2*ω*) processes of fundamental lasing modes at *λ*_1_ (1088 nm)_,_ *λ*_2_ (1074 nm), and *λ*_4_ (1057 nm), respectively. *λ*_6_ (540.5 nm) is from SFG of fundamental lasing modes of *λ*_1_ and *λ*_2_ (*ω*_1_+*ω*_2_) while *λ*_8_ (532.7 nm) is from SFG of fundamental lasing modes of *λ*_2_ and *λ*_4_ (*ω*_2_+*ω*_4_). Polarization directions of the far-field radiations from these fundamental lasing peaks indicate they have different transverse modes inside the NW (Supplementary information Fig. S12c). The intensity ratios among these self-frequency-conversion lasing modes shown in Fig. 5 are mainly caused by the different electric field distributions of the fundamental lasing modes participating in the corresponding second-order nonlinear process (Supplementary information Section 10). The power dependences between the fundamental lasing modes and the self-frequency-conversion lasing modes are discussed in detail in Supplementary information Section 10, proving the corresponding second-order nonlinear processes. Note, the three-wave-mixing processes of these fundamental lasing modes also include their DFGs and SPDCs, which could generate signals at the mid-infrared wavelength range. For example, the corresponding DFG of *λ*_2_ and *λ*_4_ (*ω*_2_ − *ω*_4_) is expected at the wavelength of 6678 nm, which could provide a pathway to realize nanoscale mid-infrared and THz coherent sources. Unfortunately, this wavelength range is beyond our instrument limits, and cannot be presented here. In addition, the generality of the self-frequency-conversion process in NW lasers is verified by measuring the fundamental lasing modes and self-frequency-conversion lasing modes from other NWs with different geometry parameters, as shown in Supplementary information Fig. S10.

## Discussion

In this work, we have demonstrated III–V compound semiconductor NW lasers could operate in a self-frequency-conversion mode relying on their strong intrinsic second-order nonlinearity, which provides a new avenue to expand the output wavelength of NW lasers beyond its gain spectral range. From a GaAs/In_0.16_Ga_0.84_As core/shell NW laser with a fundamental lasing mode at 1016 nm, a frequency-doubled lasing mode at 508 nm is obtained. Both of the lasing modes show different polarization behaviors. From another NW laser with a larger diameter, fundamental multimode lasing is observed, and importantly, these modes lead to other self-frequency-conversion laser wavelengths via SHG and SFG. Due to the severe absorption of the axial propagation of SHG and SFG in these NWs, the strategies of phase-matching and double resonance can not be utilized to improve the conversion efficiency in these frequency upconversion processes. An alternative approach is placing NW on an insulating-gap-on-metal substrate, which could form a hybrid plasmonic gap mode^37,38^. The SHG or SFG lasing mode could be guided axially along the gap mode to form a guiding mode, which is possible to reduce the reabsorption. And the phase-matching/double resonance could be achieved to further improve the SHG or SFG conversion efficiency. Although the lasing modes at the long-wavelength range supported by the self-DFG are not observed due to our limited measurement instruments, the concept of self-frequency-conversion NW laser could be applied to develop nanoscale coherent mid-infrared and THz light sources. In addition, since there is no reabsorption in the DFG process, the phase-matching and double resonance could be achieved for the lasing modes through engineering the NW geometry, which could boost the conversion efficiency and application possibility in this frequency downconversion process.

## Materials and methods

### Optical experiments

A confocal photoluminescence microscopy system (shown in Supplementary information Fig. S1) was used for the optical characterization of the NWs. A femto-second laser (Spectra-physics Mai Tai) with a central wavelength at 800 nm, pulse duration of 35 fs, and repetition rate of 85 MHz was used as the pump source. A half-wave plate (HWP) and a polarization beam splitter (PBS) were used to adjust the pump power. An objective lens (Mitutoyo M Plan ×50 NA = 0.42) was utilized to focus the pump laser on the NW and collect the emission signals from the NW as well as the reflected pump laser. The collected optical signals were transmitted through a linear polarizer to analyze the polarization states of the emission signals. A dichroic mirror, which could transmit signals with wavelengths above 932 nm and reflect signals with wavelengths below 872 nm, was placed in the light path to separate spontaneous emission and fundamental lasing light (around the wavelength of 1000 nm) from the reflected pump laser and the frequency-doubled lasing light (around the wavelength of 500 nm). The spontaneous emission or fundamental lasing light was finally analyzed by a spectrometer (Acton SpectroPro SP-2500) mounted with an InGaAs camera (PyLoN-IR) or imaged on a silicon CCD (The Imaging Source Silicon CCD DMK). The frequency-doubled lasing light was filtered from the reflected pump laser by a short-pass filter, which was then dispersed by a spectrometer into a silicon camera (PIXIS Silicon CCD).

## Supplementary information

Supplementary information for Self-frequency-conversion nanowire lasers
