# Supplementary material for: Self-frequency-conversion nanowire lasers
Source: Light Sci Appl. 2022 Apr 29;11:120. doi: 10.1038/s41377-022-00807-7 (PMC9054850; doi:10.1038/s41377-022-00807-7)
Supplement: Supplementary file 1 — Supplementary information for Self-frequency-conversion nanowire lasers [file 41377_2022_807_MOESM1_ESM.docx]

**Supplementary information for**

**Self-frequency-conversion nanowire lasers**

Ruixuan Yi^†^, Xutao Zhang^†β*^, Chen Li^†^, Bijun Zhao^†^, Jing Wang^†^, Zhiwen Li^†^, Xuetao Gan^†*^, Li Li^ξ^, Ziyuan Li^ξ^, Fanlu Zhang^ξ^, Liang Fang^†^, Naiyin Wang^ξ^, Pingping Chen^ψ§^, Wei Lu^ψ§α^, Lan Fu^ξ@^, Jianlin Zhao^†^, Hark Hoe Tan^ξ@^, and Chennupati Jagadish^ξ@^

*^†^Key Laboratory of Light Field Manipulation and Information Acquisition, Ministry of Industry and Information Technology, and Shaanxi Key Laboratory of Optical Information Technology, School of Physical Science and Technology, Northwestern Polytechnical University, Xi’an 710129, China*

*^β^Frontiers Science Center for Flexible Electronics, Xi’an Institute of Flexible Electronics (IFE) and Xi’an Institute of Biomedical Materials & Engineering, Northwestern Polytechnical University, 127 West Youyi Road, Xi'an 710072, China*

*^ξ^Department of Electronic Materials Engineering, Research School of Physics, The Australian National University, Canberra, ACT 2601, Australia.*

*^ψ^State Key Laboratory for Infrared Physics,* *Shanghai Institute of Technical Physics, Chinese Academy of Sciences,* *500 Yutian Road, Shanghai 200083, China*

*^§^University of Chinese Academy of Sciences,* *19 Yuquan Road, Beijing* *100049, China*

*^α^School of Physical Science and Technology, ShanghaiTech University, 393 Middle Huaxia Road, Pudong District, Shanghai 201210, China.*

*^@^ARC Centre of Excellence for Transformative Meta-Optical Systems, Research School of Physics, The Australian National University, Canberra, ACT 2601, Australia.*

*Email: xtzhang@nwpu.edu.cn; xuetaogan@nwpu.edu.cn

[**1. Nanowire growth method 3**](#_Toc95941829)

[**2. Schematic diagram of the optical experiment setup 3**](#_Toc95941830)

[**3. Threshold gain calculation 4**](#_Toc95941831)

[**3.1 Guided modes 4**](#_Toc95941832)

[**3.2 Confinement factor 5**](#_Toc95941833)

[**3.3 Reflectivity 6**](#_Toc95941834)

[**3.4 Propagation losses 6**](#_Toc95941835)

[**4. Euler matrix 7**](#_Toc95941836)

[**5. Second-harmonic generation collection efficiency 9**](#_Toc95941837)

[**6. Second-harmonic generation of NW pumped by an external laser 11**](#_Toc95941842)

[**7. Simulations of polarization dependence 12**](#_Toc95941843)

[**7.1 Fundamental lasing mode 12**](#_Toc95941844)

[**7.2 Frequency-doubled lasing mode 13**](#_Toc95941845)

[**8. Self-frequency-conversion in the multimode NW laser 15**](#_Toc95941846)

[**9. Reabsorption of SHG lasing mode in the NW 16**](#_Toc95941852)

[**10. Self-frequency-conversion in the multimode NW laser 18**](#_Toc95941853)

[**11. Reference: 22**](#_Toc95941854)

# Nanowire growth method

GaAs/InGaAs core/shell nanowires (NWs) were grown on GaAs (111)B substrates via selective area metal-organic vapor phase epitaxy in an AIXTRON 200/4 reactor. Before growth, we first deposited ~30 nm thick SiO_2_ film on the GaAs (111)B substrates at 300 °C by plasma-enhanced chemical vapor deposition. Then we patterned hexagonal arrays of holes through electron beam lithography and reactive ion etching. After finishing trim etching with sulfuric acid and hydrochloric acid, we loaded the substrates into the growth chamber. Trimethylgallium, trimethylindium, and arsine were used as precursors for the Ga, In, and As, respectively.

The optimized growth conditions of the GaAs/InGaAs core/shell NWs were as follows:

| Growth Step | TMGa (mol/min) | TMIn (mol/min) | AsH_3_ (mol/min) | Time | T  (°C) |
| --- | --- | --- | --- | --- | --- |
| GaAs core | 1.006E-05 | 0 | 1.998E-04 | 20 min | 750 |
| InGaAs shell | 1.006E-05 | 2.529E-06 | 4.024E-04 | 15 min | 750 |

# Schematic diagram of the optical experiment setup


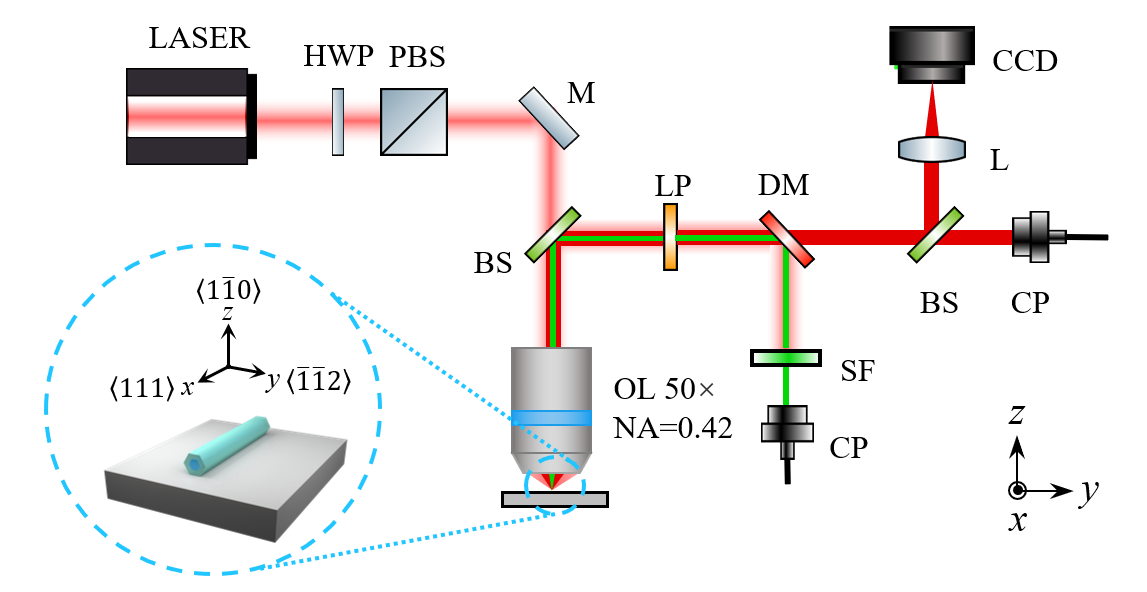


**Figure S1.** Schematic sketch of the experiment setup for measuring NW’s fundamental lasing mode and frequency-doubled lasing mode. The zoomed dashed circle shows the crystal direction of the NW with respect to the lab frame. HWP, half-wave plate; PBS, polarization beam splitter; LP, linear polarizer; M, mirror; BS, beam splitter; OL, objective lens; DM, dichroic mirror; SF, short-pass filter; CP, Collimation packages; L, lens.

# Threshold gain calculation

In this study, to identify the lasing mode of the NW lasers, we modeled the threshold gain for the core/shell GaAs/InGaAs NW lasers. The threshold gain spectra for different guided modes are shown in **Figure S3**d. And they were calculated by:^1,2^

$$g_{th}=\frac{1}{\Gamma}(\frac{1}{L}\ln\frac{1}{R}+\alpha_{i}) (1)$$

in which Γ is the confinement factor, *L* is the length of NW, *R* is the end facet reflectivity and *α*_i_ is the propagation loss. By calculating each factor via commercial software (FDTD lumerical Inc. and MODE lumerical Inc.), the theoretical *g_th_* could be estimated. As the experiment was carried out in a cryostat around 10 K, all following numerical simulations employed the refractive index of GaAs at 40 K, which is the closest temperature to 10 K from the reported value.^3^

## 3.1 Guided modes

The guided modes supported in the GaAs/InGaAs core/shell NWs were numerically calculated through MODE Solutions, Lumerical Inc. Because of the approximate refractive index of In_0.16_Ga_0.84_As and GaAs, we modeled the NW as a pure GaAs hexagonal prism (diameter as 410 nm, length as 4.4 μm), lying on a SiO_2_ substrate. Modes were calculated at 1016 nm, which is the lasing wavelength of the NW laser. The mode whose effective index is smaller than 1.45 (refractive index of substrate) is supposed to be weakly confined in NW and not guided. **Figure S2** shows electric field intensity profiles of the guided modes in the as-mentioned NW models.


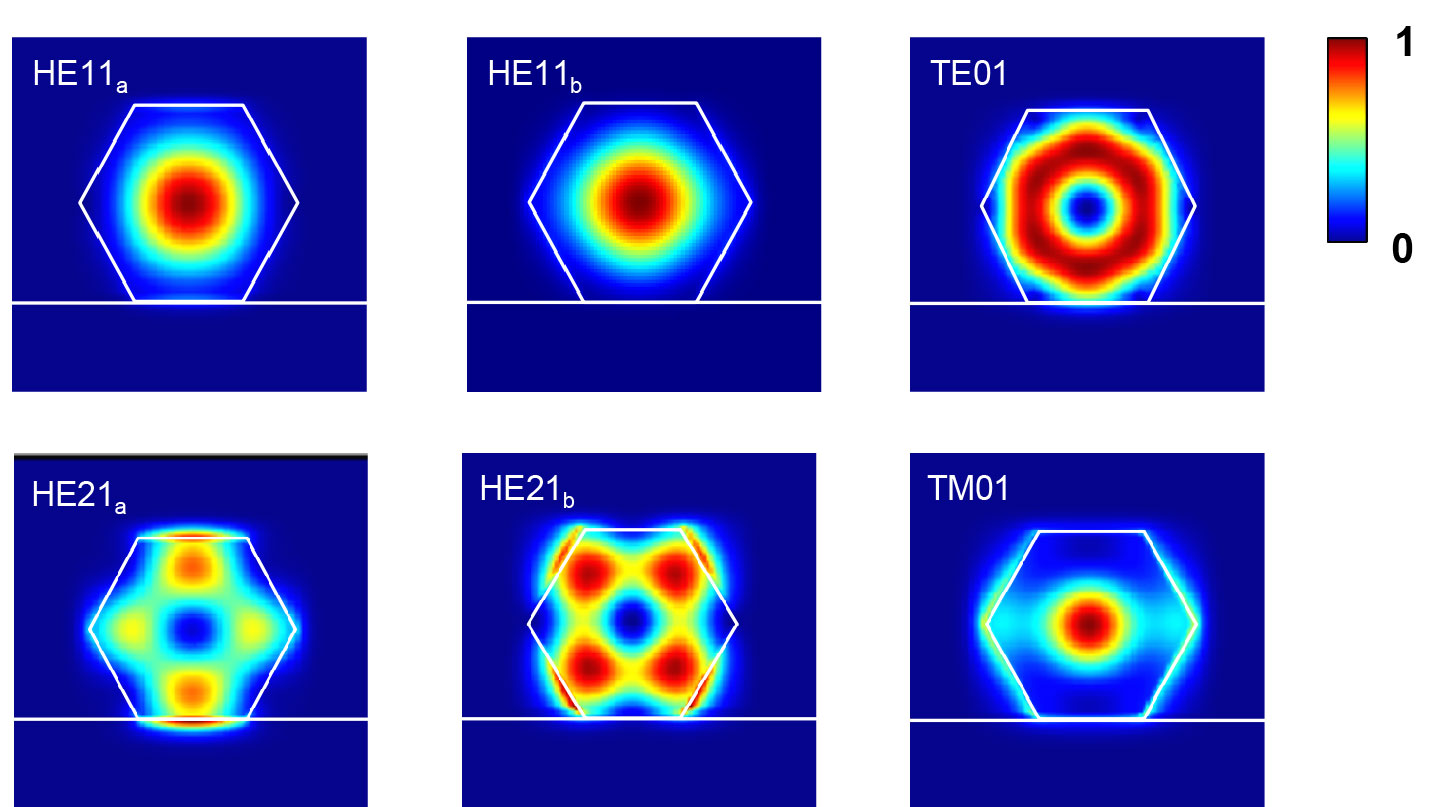


**Figure S2.** Electric field intensity profiles of different guided modes for a GaAs NW with a diameter of 410 nm lying on SiO_2_ substrate at the wavelength of 1016 nm.

## 3.2 Confinement factor

Confinement factor describes the overlap between the gain medium and optical field. It could be calculated numerically in MODE Solutions, Lumerical Inc. with the determined field profile through the formula:^1^

$$\Gamma=\frac{c\varepsilon_{0}n\iint_{a\mathrm{ctive}} \frac{1}{2}\left| \mathbf{E} \right|^{2}dxdy}{\iint_{\mathrm{waveguide}} \frac{1}{2}\mathrm{Re}[\mathbf{E}\mathbf{H}^{*}]\hat{z}dxdy} (2)$$

where **E** and **H** are the complex electric and magnetic fields of the guided modes, *c* is the light speed in vacuum, *ε*_0_ is the vacuum permittivity, and *n* is the refractive index of gain medium. The integration in numerator is over the active region of NW laser, which is the InGaAs shell in this study. The integration in dominator is over the entire simulation span. The confinement factor could also be re-written as:

$$\Gamma=\frac{n_{g}}{n}\frac{c\varepsilon_{0}n\iint_{\mathrm{active}} w_{e} dxdy}{\iint_{\mathrm{waveguide}} \frac{1}{2}\left( w_{e}+w_{m} \right)dxdy}=\frac{n_{g}}{n}\Gamma_{0} \left( 3 \right)$$

where *w*_e_ and *w*_m_ are the energy density in the electric and magnetic fields, *n*_g_ is the group index of the supported mode and Γ_0_ is the ratio of energy in the active region to the energy in the entire NW waveguide. The group index could be calculated through:

$$n_{g}=n_{\mathrm{eff}}-\lambda\frac{dn_{\mathrm{eff}}}{d\lambda} (4)$$

where *n*_eff_ is the effective index of the guided mode. **Figure S3**a shows the confinement factor versus wavelength for each guide mode.

## 3.3 Reflectivity

The reflectivity at NW/air interface was calculated through a 3D finite-difference time-domain (FDTD) simulation for each mode.^4^ In the 3D FDTD simulation, guided mode propagates along the NW and gets reflected at the NW/air interface. A power monitor was used to record the reflected optical fields. As some other modes could be excited by the reflection, the reflectivity was calculated by the overlap of reflected optical fields and injection original optical mode. **Figure S3**b shows reflectivity versus wavelength for each guided mode. Due to diffraction, the calculated facet reflectivity of high-order mode such as TE01 shown in **Figure S3**b is greater than that calculated by the Fresnel equation, which is (*n*-1)/(*n*+1) = 0.54.

## 3.4 Propagation losses

As the NW lies on substrate, when transverse mode propagates along the NW, the optical field will couple to the substrate evanescently. This part of energy attenuation could be described as propagation losses.^2^ We build a 3D FDTD model to calculate the propagation losses. In the model, a NW lied on a SiO_2_(285 nm)/Si substrate. Each transverse mode was launched and propagated along the NW. A power monitor which is 5 μm away from the mode source was used to record the transmission.

Then, the propagation losses *α*_i_ could be calculated through:

$$\alpha_{i}=-\frac{\ln\left( T \right)}{d} (5)$$

where *T* is the transmission recorded by the power monitor and *d* is the propagation distance from mode source and the power monitor. **Figure S3**c shows the propagation losses versus wavelength for each guided mode.


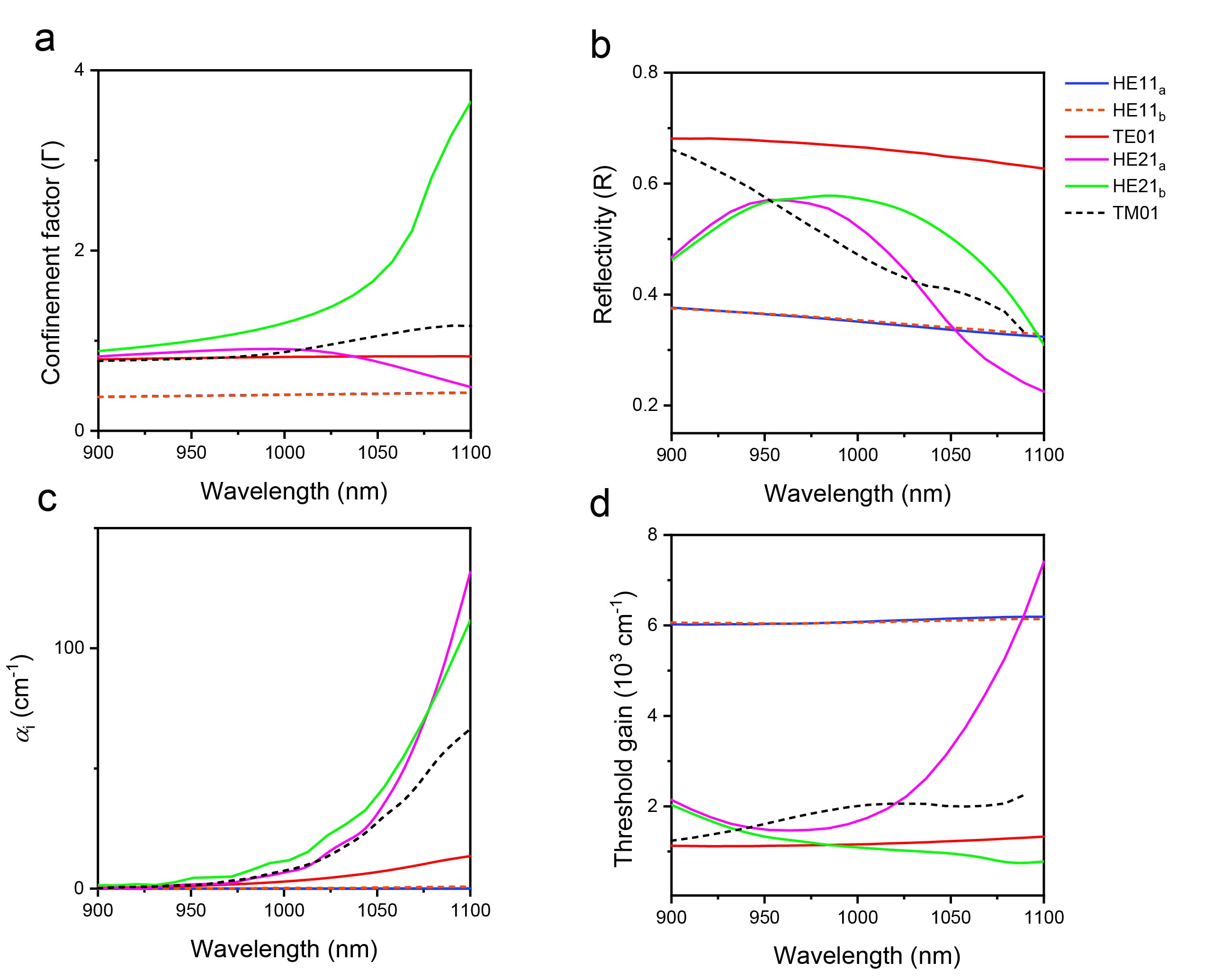


**Figure S3.** Calculated confinement factor (a), reflectivity (b), propagation losses (c) and threshold gain (d) as a function of wavelength for different guided modes of the GaAs/InGaAs core/shell NW lying on the SiO_2_ substrate.

# Euler matrix

To calculate the second-order nonlinear polarization of zincblende GaAs through equation:

$$\mathbf{P}_{c}=2\varepsilon_{0}\left[ \begin{matrix} 0 & 0 & 0 & d_{14} & 0 & 0 \\ 0 & 0 & 0 & 0 & d_{25} & 0 \\ 0 & 0 & 0 & 0 & 0 & d_{36} \end{matrix} \right]\left[ \begin{matrix} E_{cx}^{2} \\ E_{cy}^{2} \\ E_{cz}^{2} \\ {2E}_{cy}E_{cz} \\ {2E}_{cx}E_{cz} \\ {2E}_{x}E_{y} \end{matrix} \right] (6)$$

, the excited electric field should be defined in the crystal frame in **Figure S4**a. As shown in **Figure S4**b, the lab frame does not coincide with the crystal frame in this study. The excited electric fields which are extracted from FDTD are defined in the lab frame. An Euler matrix **R** could be utilized to transform electric field components from the lab frame into the crystal frame through a matrix multiplication:^5,6^

$$\left[ \begin{matrix} E_{cx} \\ E_{cy} \\ E_{cz} \end{matrix} \right]=\mathbf{R}\left[ \begin{matrix} E_{x} \\ E_{y} \\ E_{z} \end{matrix} \right]=\left[ \begin{matrix} \cos\left( \alpha\right)\cos\left( \gamma\right)-\cos\left( \beta\right)\sin\left( \alpha\right)\sin\left( \gamma\right) & -\cos\left( \alpha\right)\sin\left( \gamma\right)-\cos\left( \beta\right)\cos\left( \gamma\right)\sin\left( \alpha\right) & \sin\left( \alpha\right)\sin\left( \beta\right) \\ \cos\left( \gamma\right)\sin\left( \alpha\right)+\cos\left( \alpha\right)\cos\left( \beta\right)\sin\left( \gamma\right) & \cos\left( \alpha\right)\cos\left( \beta\right)\cos\left( \gamma\right)-\sin\left( \alpha\right)\sin\left( \gamma\right) & -\cos\left( \alpha\right)\sin\left( \beta\right) \\ \sin\left( \beta\right)\sin\left( \gamma\right) & \cos\left( \gamma\right)\sin\left( \beta\right) & \cos\left( \beta\right) \end{matrix} \right]$$

$$\left[ \begin{matrix} E_{x} \\ E_{y} \\ E_{z} \end{matrix} \right] (7)$$

where *α*, *β*, *γ* are the Euler angles defined in **Figure S4**c. In this study, according to the determined crystal direction along the lab frame, the Euler angle could be solved as *α* = 144.7˚, *β* = 90˚, *γ* = 45˚.

**
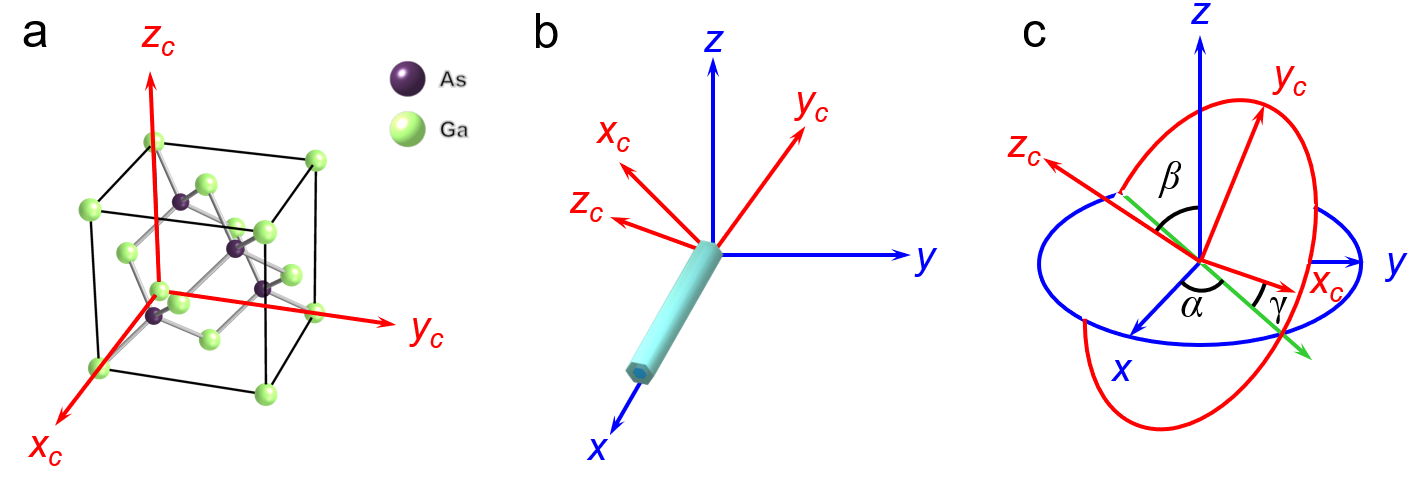
**

**Figure S4.** a. Crystal frame (*x*_c_*y*_c_*z*_c_) defined under crystal structure. b. Schematic of the lab frame (*xyz*) and the crystal frame (*x*_c_*y*_c_*z*_c_). c. Geometry of the rotation from the crystal frame to the lab frame.

# Second-harmonic generation collection efficiency

The second-order nonlinear polarization could be regarded as electric dipoles oscillate at 2*ω* and radiate second-harmonic generation (SHG) electromagnetic wave into free space. As electric dipole does not radiate simple spherical wave, it is necessary to consider the collection efficiency of each polarization component for accurate estimation of SHG intensity. Here, we calculated the collection efficiency of the objective lens through 3D FDTD simulations for in-plane and out-of-plane second-order nonlinear polarization.

**Figure S5** shows corresponding simulation setups in FDTD. The electric dipole was set above the SiO_2_ substrate with a height of 200 nm, and a 2D monitor was set above the dipole with a height of 400 nm to record the near-field.


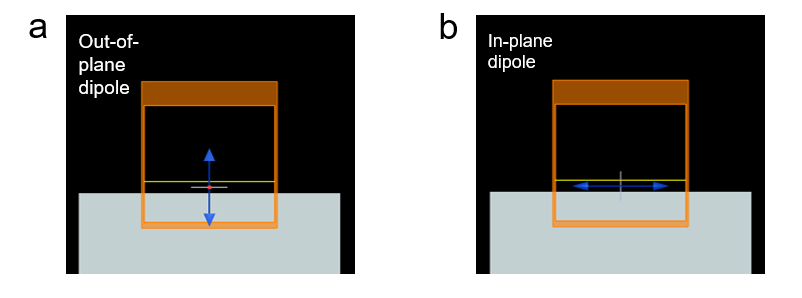


**Figure S5**. Simulation setups for numerically calculating the collection efficiency of objective lens for out-of-plane (a) and in-plane (b) electric dipoles radiation in FDTD.

As shown in **Figure S6**a, due to the limited numerical aperture (NA) of the objective lens, only part of SHG whose propagation direction is in a range determined by NA would be collected by the objective lens. In FDTD simulation, far-field is a decomposition of the near-field using a set of plane waves propagating at different angles as the basis for decomposition. Therefore, far-field data could be used to calculate the impact of limited NA on collection efficiency.


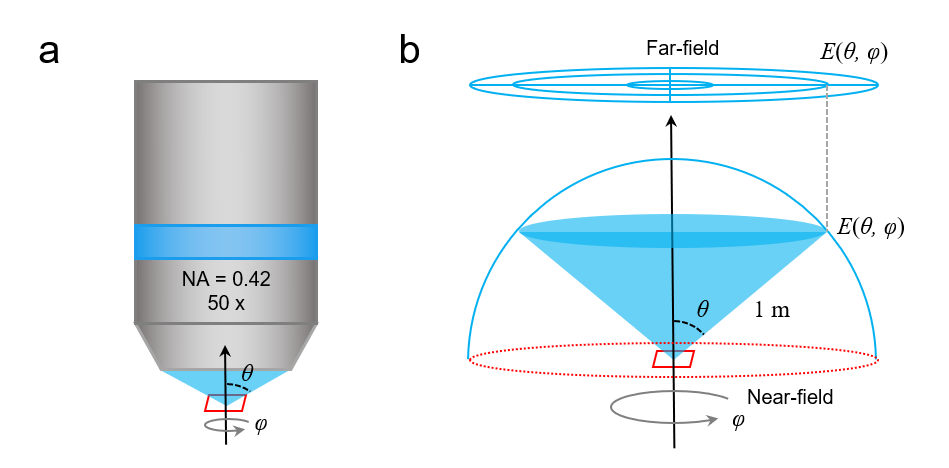


**Figure S6.** a. The objective lens could only collect limited signal which is in a specific range determined by NA. b. The far-field is numerically calculated by launching the near-field data recorded by the monitor (red rectangle) to a hemisphere 1 meter away.

The far-field could be numerically calculated from the near-field data through the process described in **Figure S6**b. The ratio of collected signal power to the total emission power is collection efficiency *η*, which could be calculated through:

$$\eta=\frac{\int_{0˚}^{\theta_{0}} \int_{0˚}^{\varphi_{0}} E^{2}sin(\theta)d\theta d\varphi}{\int_{0˚}^{90˚} \int_{0˚}^{360˚} E^{2}sin(\theta)d\theta d\varphi} (8)$$

where *E*^2^ is the electric field intensity in the far-field profile, and the integration limit of numerator, $\theta_{0}$ and $\varphi_{0}$ are determined by the NA of the employed objective lens.


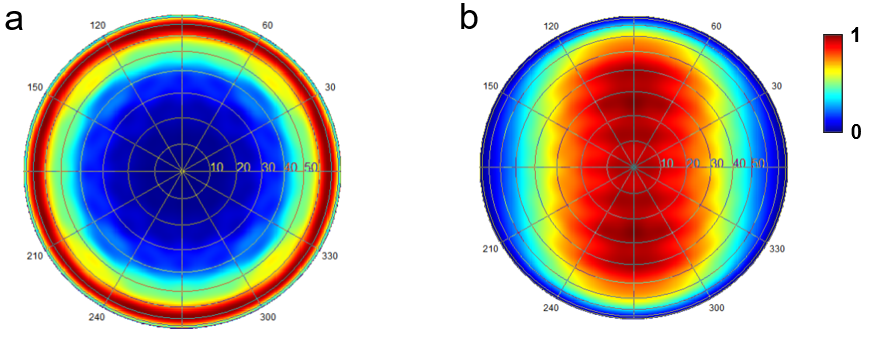


**Figure S7.** Far-field profiles (electric field intensity) for the in-plane and out-of-plane electric dipoles.

As shown in **Figure S7**, the far-field radiation pattern of the in-plane dipole is like a solid ellipse while the far-field radiation pattern of the out-of-plane dipole is like a donut. It means that majority of radiation from out-of-plane dipole could not be collected by normal objective lens effectively. For an objective lens with NA = 0.42, the ratio of *η*_out-of-plane_ (*P_z_*) to *η*_in-plane_ (*P_x_* and *P_y_*) is 0.01. So, SHG which is generated by the out-of-plane polarization is negligible compared to in-plane polarization and *η*_out-plane_ ≈ 0. And we only consider in-plane polarization radiating SHG in this study.

# Second-harmonic generation of NW pumped by an external laser

SHG process of the NW is confirmed further by pumping it with an external ultrafast laser. As shown in **Figure S8**a, a signal peak at 400 nm was observed, which is regraded as the SHG signal of the pump laser at 800 nm. A pump polarization dependence of the SHG signal was carried out. In this experiment, the polarization direction of pump laser was continuously changed by rotating a half-wave plate and the corresponding intensities of the SHG peak were recorded (shown as blue dots in **Figure S8**b). According the crystal direction determined by TEM analysis, Euler angle was determined as *α* = 144.7˚, *β* = 90˚, *γ* = 45˚. By using Equations (6) and (7) and ignoring *P*_z_, we calculated the theoretical results for the above experiment (shown as blue line in **Figure S8**b). The theoretical results match with experimental results. On the one hand, it confirms the peak at 400 nm is SHG of pump laser. On the other hand, it proves the correctness of Euler angles determined by TEM analysis, which is fundamental for the calculation of the second-order nonlinear polarization based on the fundamental transverse mode.


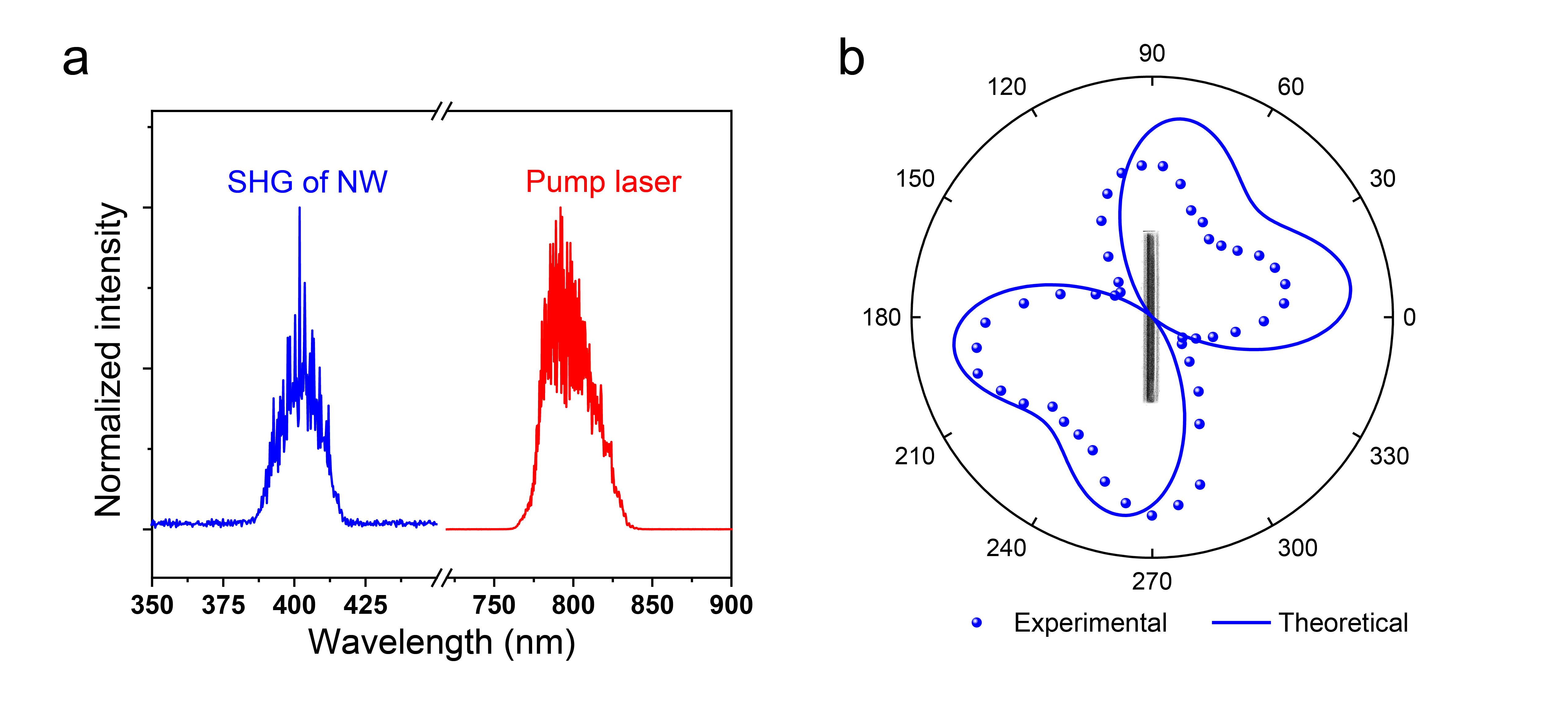


**Figure S8.** a. Spectra of the pump laser and SHG signal. b. Experimental (blue dots) and theoretical (bule line) results of the SHG response as a function of the polarization direction of the pump laser.

# Simulations of polarization dependence

## 7.1 Fundamental lasing mode

3D FDTD simulations were performed to identify the lasing mode of the NW. In order to finally determine the lasing mode of the NW, we performed 3D FDTD simulations.^7^ A simplified model, a GaAs NW lying on a SiO_2_ substrate, was used in the simulation. A 2D field monitor which can record the near-field distribution was placed at a height of 200 nm above the top surface of NW. The far-field data could be directly calculated from the near-field distribution. Because only a fraction of electromagnetic wave can be collected by the setup, the limited NA of objective lens should be taken into account. As NA of the objective lens in experiment equals to 0.42, the far-field intensity for different polarization angle, relative to the nanowire axis, was calculated first, and then integrated from 0 to 24.85° for polar angle in spherical coordinates. Then, the simulated results of the polarization dependence for each guided mode as the fundamental lasing mode are obtained and shown in **Figure S9**.

## 7.2 Frequency-doubled lasing mode

The simulation process has been described in the manuscript exhaustively. In **Figure S9**, we show the polarization dependence of frequency-doubled lasing mode for each guided mode, which is supported in the NW with a diameter of 410 nm and a length of 4.4 μm. Different frequency-doubled lasing modes show different orientation of polarization dependence. It is resulted by the specific electric field distribution of each eigenmode under the crystal frame. According to simulation results, only HE21_b_ match with the experiment data (both fundamental and frequency-doubled lasing mode).


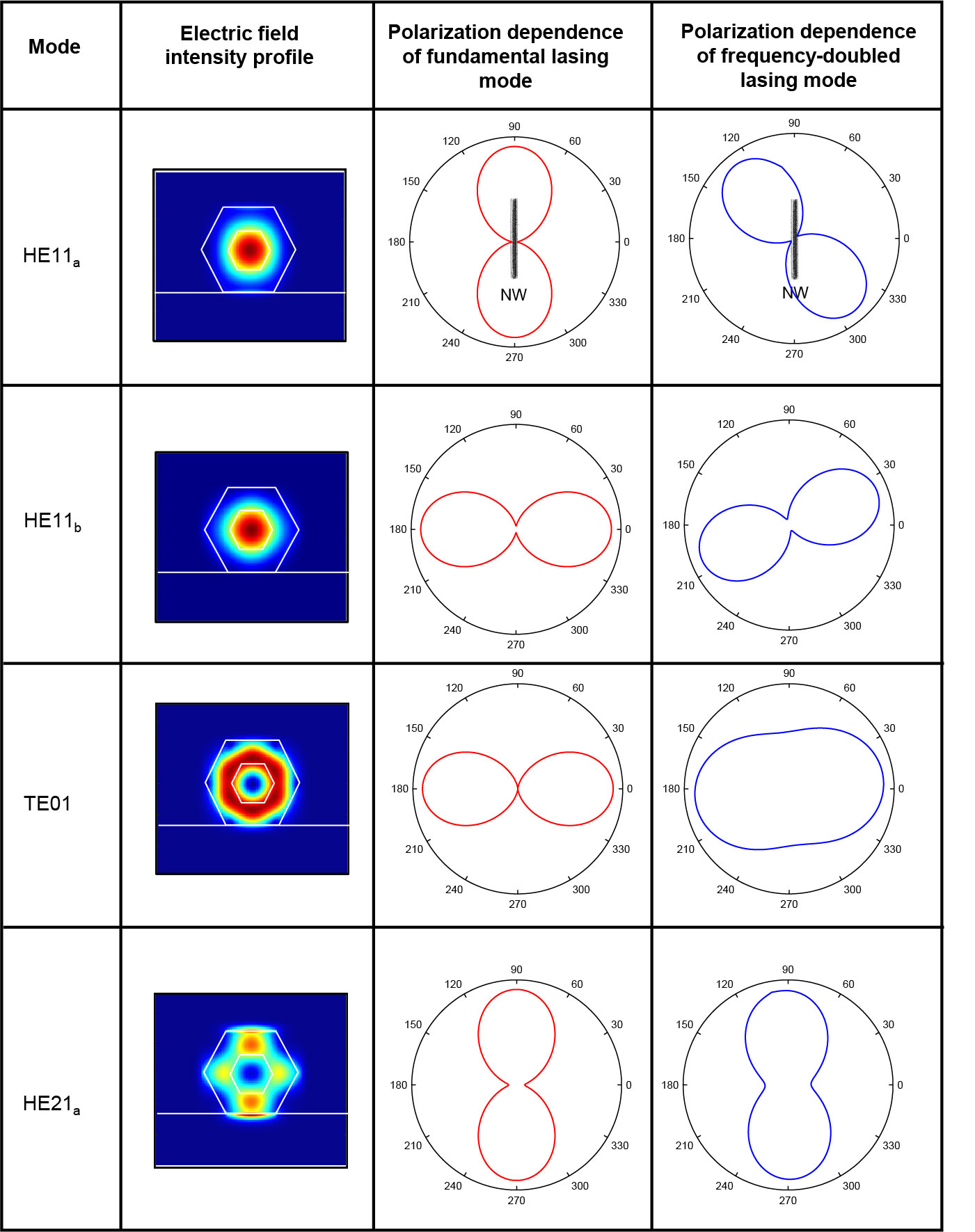


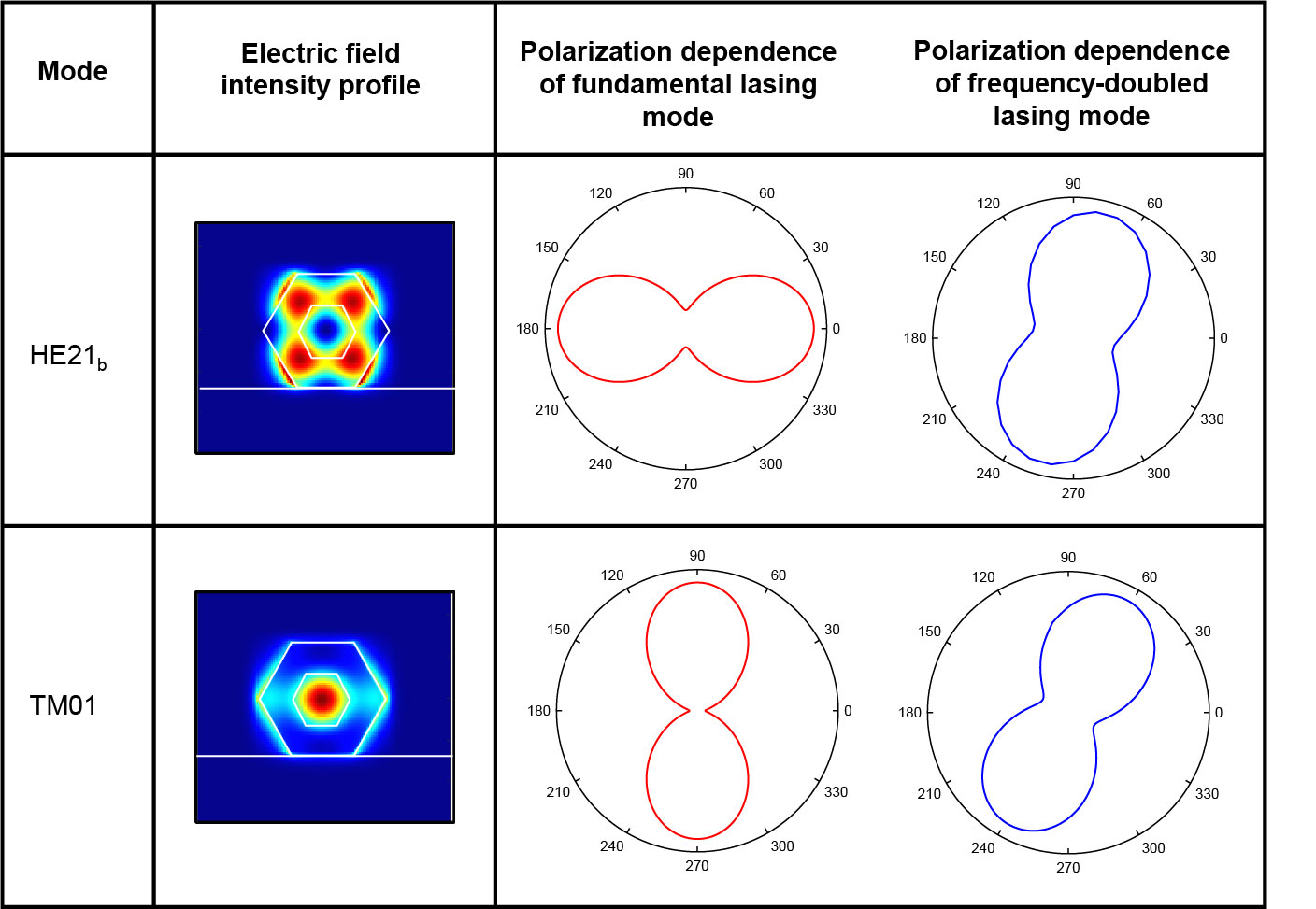


**Figure S9.** Simulation results of polarization dependence for different fundamental lasing modes and the corresponding frequency-doubled lasing modes of the NW with a diameter of 410 nm and a length of 4.4 μm. The relative orientation of NW is shown in HE11_a_ mode profile and remains unchanged.

# Self-frequency-conversion in the multimode NW laser

Self-frequency-conversion processes were observed in different NW lasers, which demonstrate the generality of self-frequency-conversion NW lasers. The lasing mode spectra and the corresponding SEM images are shown in **Figure S10**. NW 1(diameter: 465 nm, length: 4464 nm) supports a fundamental lasing mode at 1036 nm and a self-frequency-conversion lasing mode (the frequency-doubled lasing mode) at 518 nm. NW 2(diameter: 405 nm, length: 4637 nm) supports a fundamental lasing mode at 1010 nm and a self-frequency-conversion lasing mode at 505 nm. NW 3(diameter: 437 nm, length: 4610 nm) supports two fundamental lasing modes at 1010 nm (*λ*_1_) and 992 nm (*λ*_2_), and three self-frequency-conversion lasing modes at 505 nm (*λ*_3_), 500.5 nm (*λ*_4_) and 496 nm (*λ*_5_), respectively. Lasing modes at *λ*_3_ (505 nm) and *λ*_5_ (496 nm) are generated from the SHG processes of lasing modes at *λ*_1_ (1010 nm) and *λ*_2_ (992 nm), respectively. And the lasing mode at *λ*_4_ (505 nm) is SFG of lasing modes at *λ*_1_ (1010 nm) and *λ*_2_ (992nm).

**
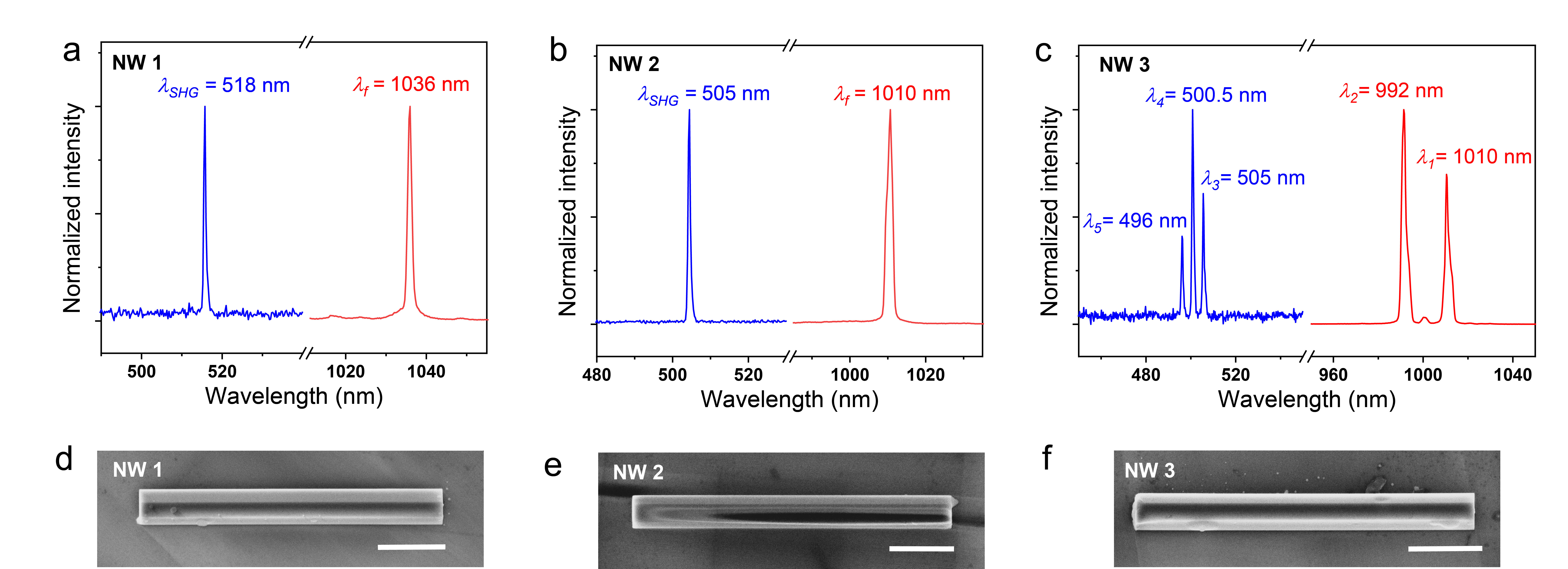
**

**Figure S10.** Normalized spectra (a-c) of the fundamental lasing mode and self-frequency-conversion lasing mode and the corresponding SEM images (d-f) of different NWs. Scale bar, 1 μm

# Reabsorption of SHG lasing mode in the NW

The SHG wavelength of the fundamental lasing mode is about 500 nm, where GaAs material has a strong absorption coefficient of 10.70 μm^-1^.^8^ The calculated absorption depth of GaAs at 500 nm is of ~ 90 nm. The axial propagation of SHG would not form a guiding mode. The measured SHG signal originates from the radiation of oscillating second-order nonlinear polarizations which directly pass through the NW surface in the normal direction. So, the distance between NW surface and second-order nonlinear polarizations, which oscillate and radiate the SHG signal, seriously affects the radiation efficiency of the SHG lasing mode outside the NW. The different transverse mode induced second-order nonlinear polarizations distribution varies greatly in space. The electric field intensity and the calculated second-order nonlinear polarizations of the donut-like transverse mode (HE21_b_) and the fundamental mode (HE11_b_) are shown in **Figure S11** for a NW with a diameter of 410 nm.


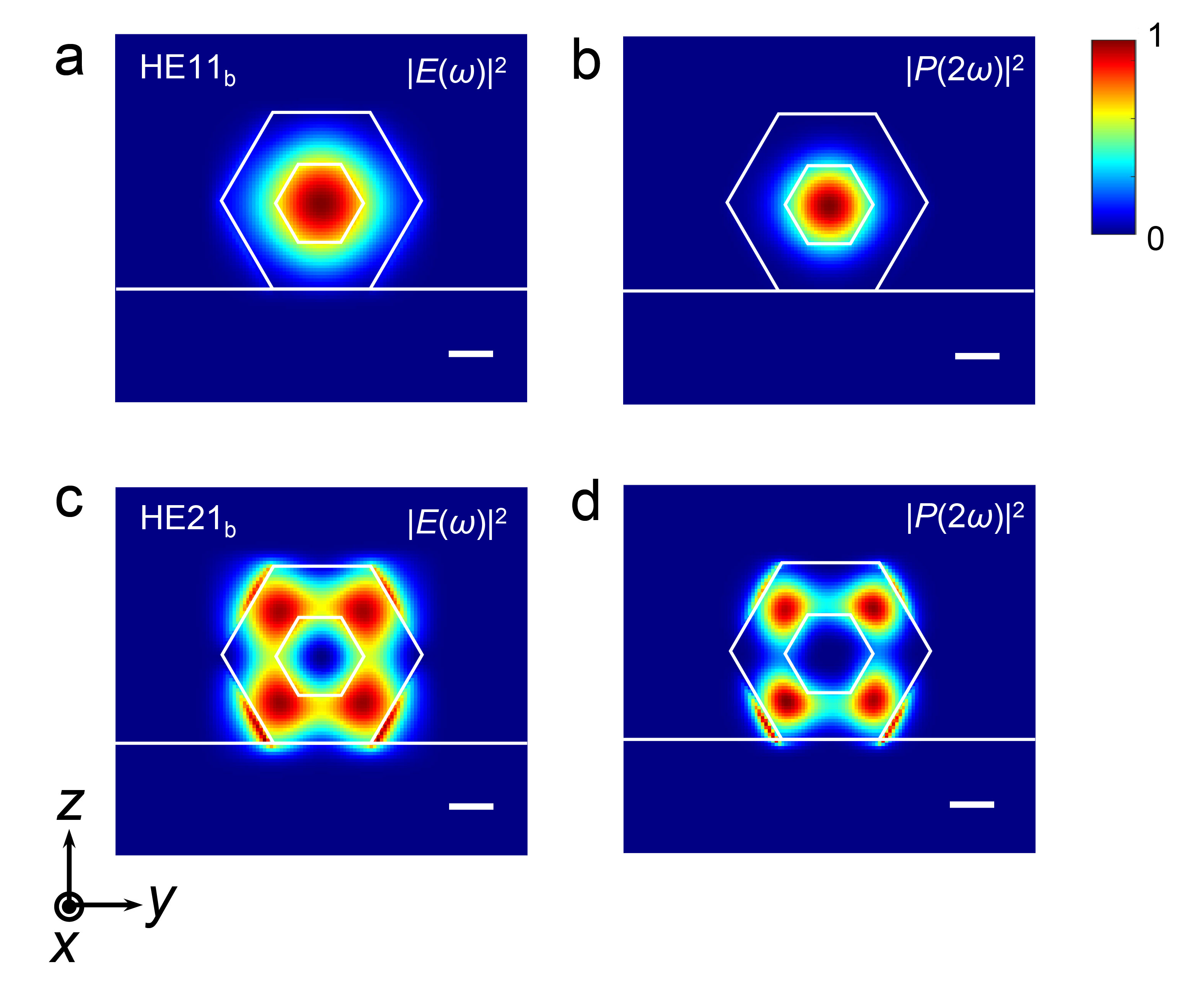


**Figure S11.** Electric field intensity profiles of the HE_11b_ (a) and HE_21b_ (c) mode. Calculated second-order nonlinear polarization of HE_11b_ (b) and HE_21b_ (d). Scale bar, 100 nm.

It indicates that, compared with second-order nonlinear polarizations induced by HE_11b_ mode, the distribution of second-order nonlinear polarizations induced by HE_21b_ mode is closer to surface of NW. For example, radiation from a dipole light source at the wavelength of 500 nm at the center of NW (distance to surface: 200 nm) will be reduced to 11% at surface. But, for the one located in the shell region (distance to surface: 50 nm), the radiation will be reduced to 58% at surface. Therefore, the second-order nonlinear polarizations of HE_21b_ will be absorbed much less than that of HE_11b_, which means a higher radiation or collection efficiency of the SHG lasing mode outside the NW.

# Self-frequency-conversion in the multimode NW laser

For a NW with diameter of 520 nm and length of 3.4 μm, it supports multiple fundamental lasing modes. Several lasing peaks resulted from second-order nonlinear effect including SHG and SFG in the visible wavelength range were generated, as illustrated in **Figure 5** of the **Maintext** and **Figure S12**a-b. The power dependent spectra of the SHG/SFG peaks and the fundamental lasing peaks are shown in **Figure S12**a. All the peaks are clearly labeled and shown in **Figure S12**b. The intensity ratios between these SHG/SFG peaks are determined by different fundamental lasing modes which participate in the second-order nonlinear process.

As reported in works about SHG of NW directly excited by an external pump laser,^6^ governed by the anisotropic second-order coefficient of zincblende GaAs, SHG intensity depends on the polarization direction of the pump laser. That is, the SHG intensity in NW is altered by the directions and distributions of the electric field in the pump laser. As shown in **Figure S12**c, the experimentally measured far-field polarization dependences of the multiple fundamental lasing modes of the NW indicate that these modes have different polarizations, which suggests these fundamental lasing peaks originated from different transverse modes with different polarizations and distributions of the electric fields. Therefore, second-order nonlinear processes of these fundamental lasing modes will generate different intensities of the frequency-conversion signals, showing intensity ratios among these self-frequency-conversion peaks in **Figure** 5 of the **Maintext** and **Figure S12**a-b.

The power dependences between the fundamental lasing modes and the self-frequency-conversion lasing modes are discussed further. By extracting peak intensities from the power dependent spectra (**Figure S12**a), a line slope of 1.68±0.01 between the fundamental lasing peak *ω*_2_ and the SHG peak *ω*_7_ was fitted in double-logarithmic axes, which could be considered as approximate quadratic function (**Figure S12**d). For the SHG peak *ω*_9_, a line slope of 1.54±0.01 was fitted versus the fundamental lasing peak *ω*_4_ (**Figure S12**e). For SFG peak *ω*_8_, due to the different lasing thresholds of the fundamental lasing modes, *ω*_2_ and *ω*_4_, the intensities of *ω*_2_ and *ω*_4_ are not proportional under different pump fluences. The SFG intensity versus the fundamental lasing peak intensity can’t be fitted with a quadratic function. We plotted the intensity *ω*_8_ versus the product intensity of *ω*_2_ and *ω*_4_, showing a fitted line slope of 0.83, which is close to the theoretical line slope of 1 according to the 2^nd^ nonlinear process (**Figure S12**f).^9^ Thence, these self-frequency-conversion peaks (*ω*_7_, *ω*_8_ and *ω*_9_) originate from second-order non-linear processes of fundamental lasing modes. The power dependences of *ω*_5_ and *ω*_6_ are not discussed here because of the comparable intensity to the spectrum noise.

The deviation of the fitted slopes from the theoretical values here could be attributed to the degradation of the NWs during the long measurements time. In the experiments, due to the large intensity difference between the self-frequency-conversion and fundamental lasing signals, they can’t be measured at the same time strictly. At a certain pump fluence, we start recording the fundamental lasing and the self-frequency-conversion signals with two spectrometers at the same time. The integral time of the camera for measuring the self-frequency-conversion signal (~5 s) is much longer than that for measuring the fundamental lasing signal (~0.1 s). During the long process of recording the self-frequency-conversion signal, the NW quality degrades and the fundamental lasing intensities gradually drop with huge number of femtosecond laser pulses pump (repetition rate of the femtosecond laser: 85 MHz). This phenomenon will be discussed in detail later. Therefore, the recorded fundamental lasing intensities in the first 0.1 s are higher than the actual intensities employed to pump the self-frequency-conversion processes in the whole 5 s. It results in that the fitted slopes are slightly lower than the theoretical values.


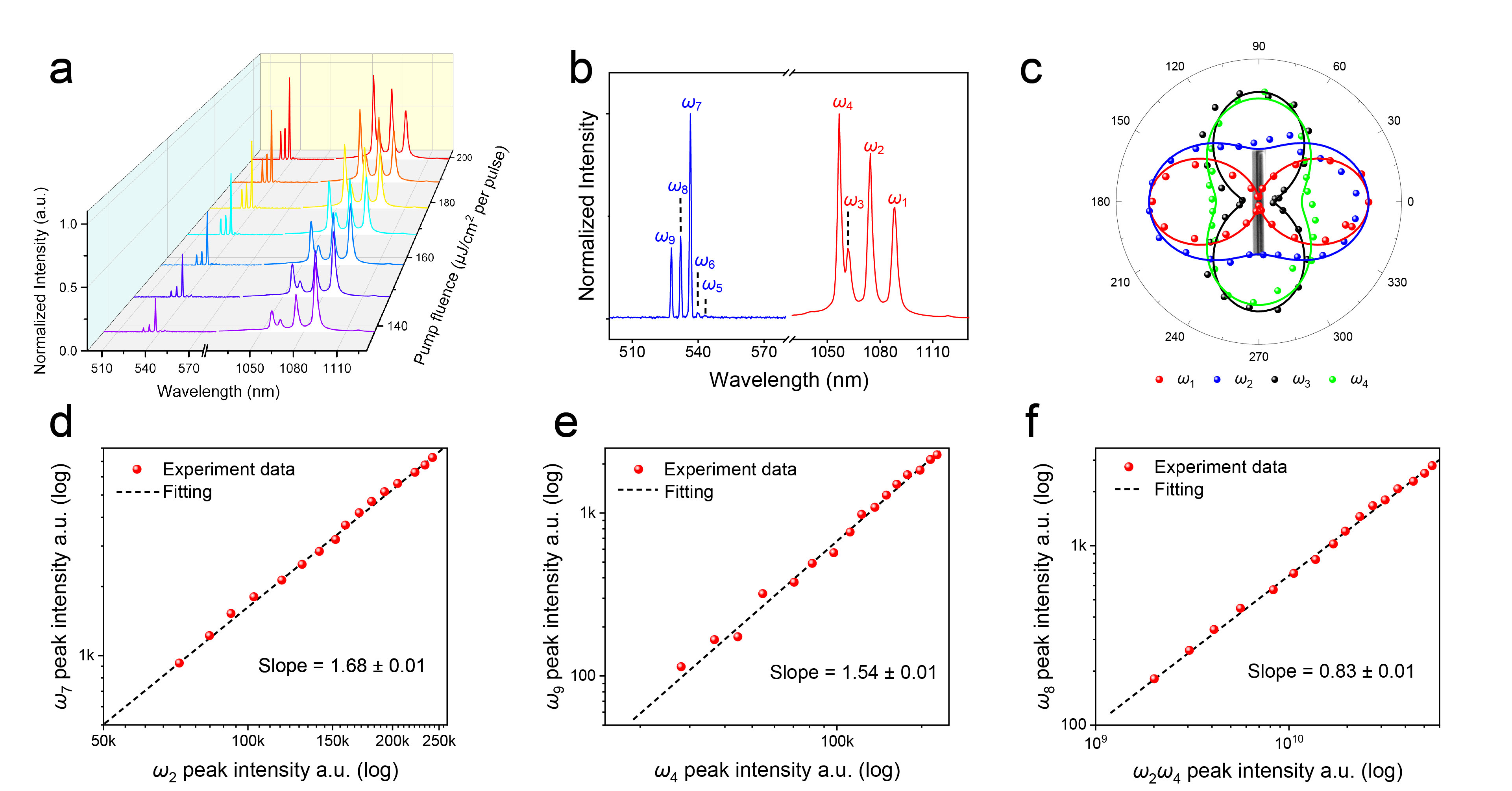


**Figure S12.** a. Normalized emission spectra of self-frequency-conversion lasing modes and fundamental lasing modes with increasing pump fluence. b. Normalized emission spectra of self-frequency-conversion lasing modes and fundamental lasing modes at a pump fluence of 199 μJ/cm^2^/pulse with labeled peaks. c. Polarization dependences of different fundamental lasing modes. d. Peak intensity dependence of *ω*_7_ versus *ω*_2_, showing a fitting slope of 1.68±0.01. e. Peak intensity dependence of *ω*_9_ versus *ω*_4_, showing a fitting slope of 1.54±0.01. f. Peak intensity dependence of *ω*_8_ versus the product the peaks of *ω*_2_ and *ω*_4_, showing a fitting slop of 0.83±0.01.

The core/shell NW lasing intensities degrade with the duration of operation under intense optical excitation. It could account for the deviation of fitting slope from theoretical value in **Figure S12**d, e and f. **Figure S13** shows the evolution of lasing spectra (a) and corresponding extracted peak intensities of Peak 1 and 2 (b, c) versus duration of excitation under a pump fluence of five times of lasing threshold for another NW. The test conditions are the same as the self-frequency-conversion experiment conditions. The red-lined spectra and red-dotted peak intensities indicate a gradual decrease in lasing intensity under continuous excitation (about 27 s). To identify the reason for the decrease of the peak intensities, the pump laser was blocked for 5 mins (300 s) to conduct the heat and cool the NW to the ambient temperature. After this, the NW was excited again under the same pump fluence. As shown by the blue-lined spectrum and blue-dotted peak intensity in **Figure S13**b and c, the collected lasing intensity did not restore to the initial value at the time of 0 s, but was close to the values obtained before blocking the pump laser (at the time of 27 s). The results from the two lasing peaks both indicate that the gradual degradation of the lasing process is irreversible. Similar photodegradation phenomena have been observed in GaAs NWs.^10,11^ Under the condition of intense laser illumination, a large number of non-radiative centers were formed in the NW, resulted by a recombination-enhanced defect reaction mechanism.^12^ The number of these centers increases with the duration of excitation. Finally, these laser-induced non-radiative centers irreversibly weaken the lasing emission efficiency, leading to a decrease in peak intensity. In addition, laser-induced heating is another possible reason to reduce the lasing efficiency and intensity. However, this process is reversible. The peak intensity could restore when the heat on NWs from the pump laser is conducted away. The results shown in **Figure S13** could exclude the effect of laser-induced heating on the degradation of lasing process.


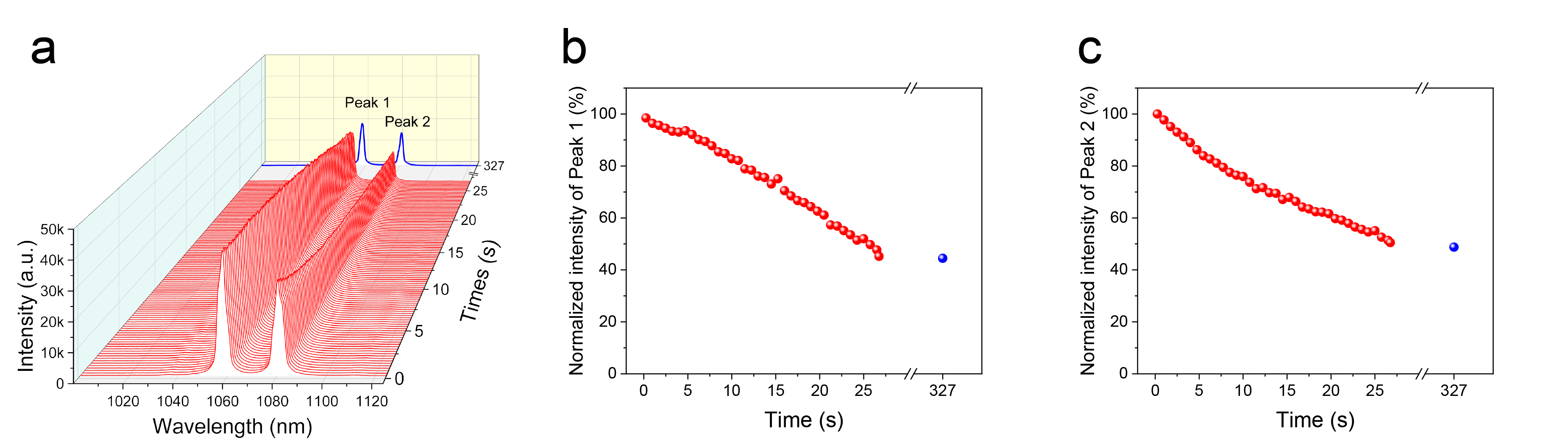


**Figure S13.** Lasing spectra (a) excited by a pump fluence of five times of lasing threshold and corresponding extracted peak intensities of Peak 1 and 2 after normalization (b, c) versus operation duration. To verify the stability and reversibility of the lasing process, the NW was first continuously excited for about 27 s. The collected spectra and corresponding peak intensities were plotted as red lines and red dots. Then, after the pump laser was blocked for 5 mins (300 s), the NW was excited again under the same condition. The collected spectrum and corresponding peak intensities were plotted as bule line and bule dots.

# Reference:

1. Ning, C. Z. Semiconductor nanolasers. *Physica Status Solidi* (*B*) **247**, 774–788 (2010).
2. Chen, S. *et al.* Dilute nitride nanowire lasers based on a GaAs/GaNAs core/shell structure. *Nano Letters* **17**, 1775–1781 (2017).
3. Gehrsitz, S. *et al.* The refractive index of Al_x_Ga_1-x_As below the band gap: Accurate determination and empirical modeling. *Journal of Applied Physics* **87**, 7825–7837 (2000).

4. Maslov, A. V. & Ning, C. Z. Reflection of guided modes in a semiconductor nanowire laser. *Applied Physics Letters* **83**, 1237–1239 (2003).

5. Hu, H. *et al.* Precise determination of the crystallographic orientations in single ZnS nanowires by second-harmonic generation microscopy. *Nano Letters* **15**, 3351–3357 (2015).

6. Timofeeva, M. *et al.* Polar second-harmonic imaging to resolve pure and mixed crystal phases along GaAs nanowires. *Nano Letters* **16**, 6290–6297 (2016).

7. Saxena, D. *et al.* Mode profiling of semiconductor nanowire lasers. *Nano Letters* **15**, 5342–5348 (2015).

8. Palik, E. D. Handbook of Optical Constants of Solids Volume III. (San Diego: Academic Press, 1998).

9. Powers, P. E. Field Guide to Nonlinear Optics. (Society of Photo-Optical Instrumentation Engineers, 2013).

10. Alekseev, P. A. *et al.* Observing visible-range photoluminescence in GaAs nanowires modified by laser irradiation. *Journal of Applied Physics* **121**, (2017).

11. Alekseev, P. A. *et al.* Photodegradation of surface passivated GaAs nanowires. *Journal of Physics Conference Series* **1461**, (2020).

12. Guidotti, D., Hasan, E., Hovel, H. J. & Albert, M. Model for degradation of band gap photo-luminescence in GaAs. *Applied Physics Letters* **11**, 583–613 (1989).
